# Supplementary figures and images for: Expression characteristics and regulatory mechanism of Apela gene in liver of chicken (Gallus gallus)
Source: PLoS One. 2020 Sep 11;15(9):e0238784. doi: 10.1371/journal.pone.0238784 (PMC7485868; doi:10.1371/journal.pone.0238784)

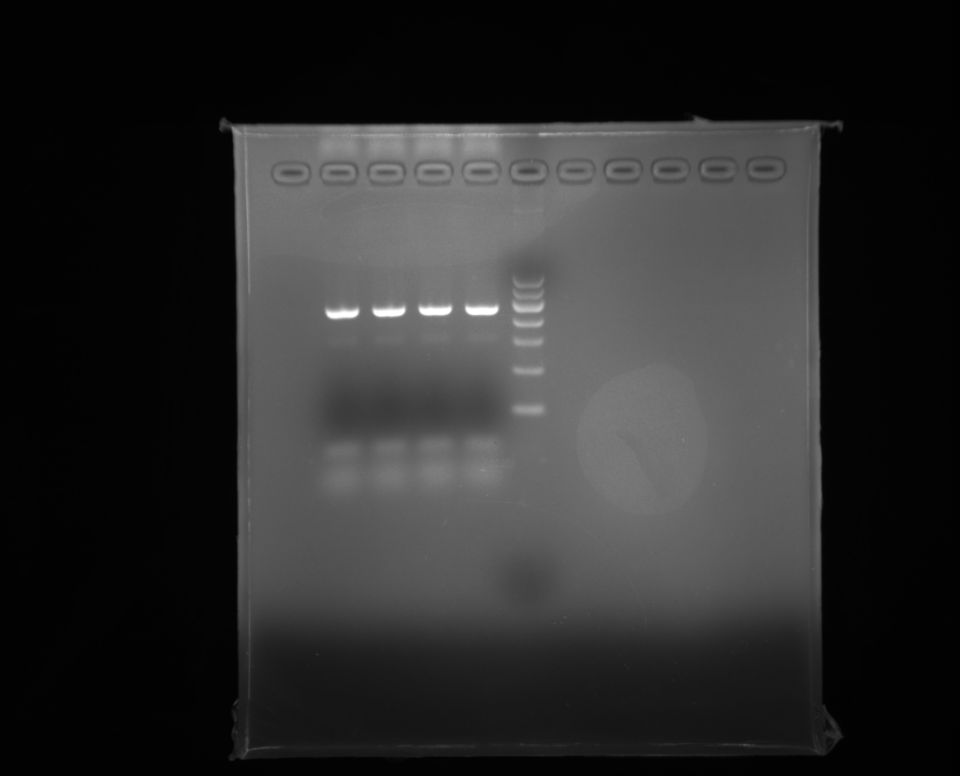

Supplement: S1 Raw image — (JPG) [file pone.0238784.s001.jpg]

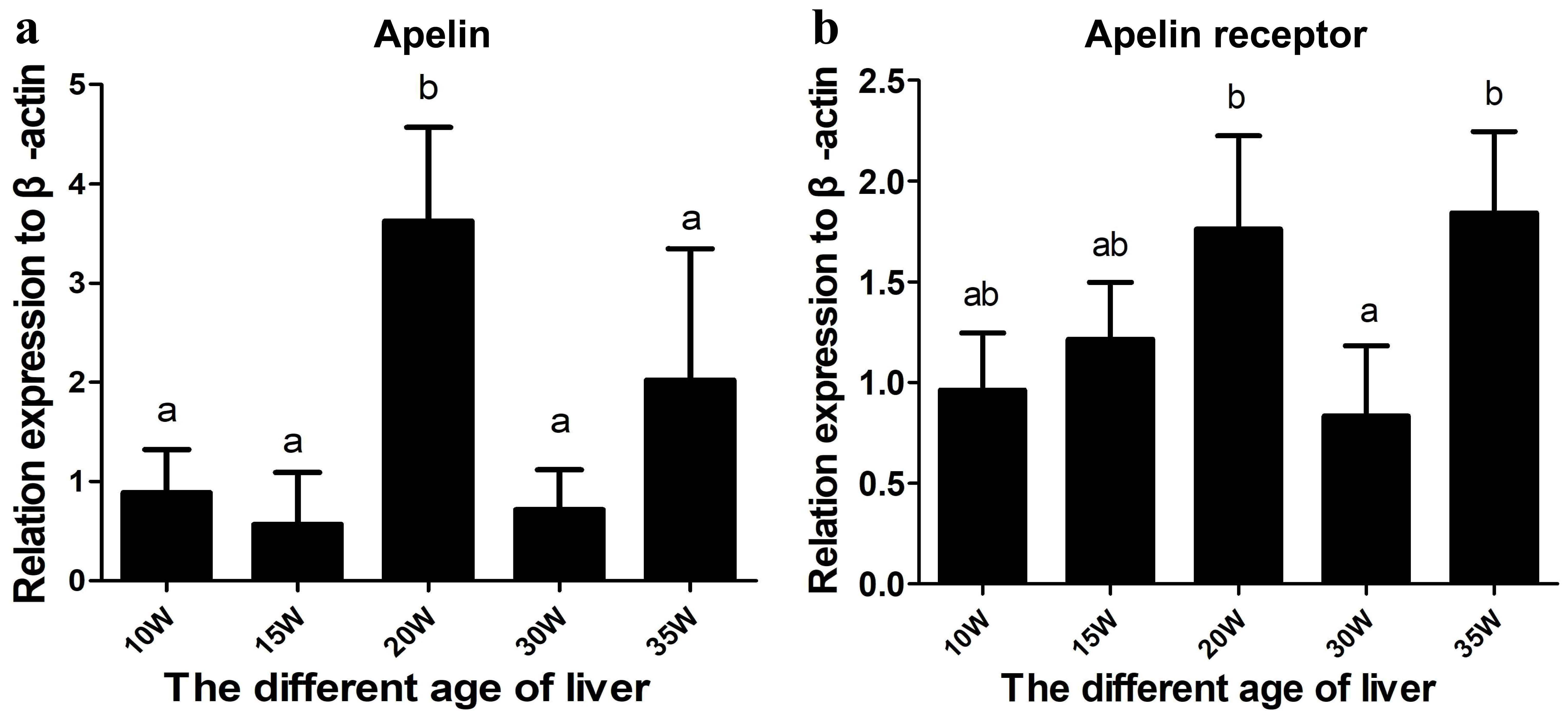

Supplement: S1 Fig — 1d means liver tissue of 1day-old chicks. 1w, 10w, 15w, 20w, 30w and 35w represent liver tissue of 1week-, 10week-, 15week-, 20week-, 30week- and 35week-old chicken, respectively. The mRNA levels of Apelin and Apelin receptor gene was normalized to the mRNA levels of β-actin. Each data point represents the mean ± SEM of 6 chicken. Different lower-case letters mean significant difference (p ≤ 0.05), and the same lower-case letter means no significant difference (p > 0.05). (TIF) [file pone.0238784.s002.tif]

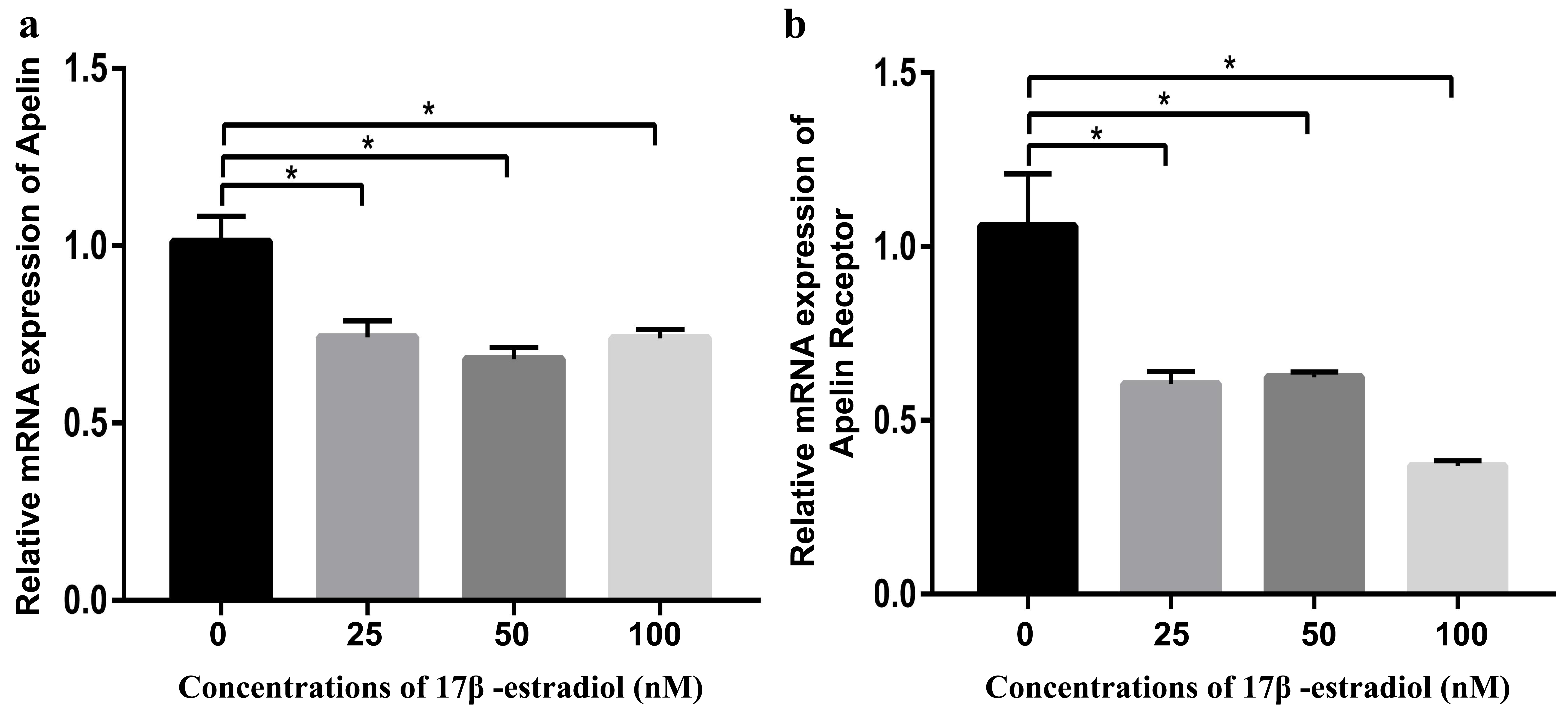

Supplement: S2 Fig — (a) The mRNA expression of Apelin in hepatocytes. (b) The mRNA expression of Apelin receptor in hepatocytes. The control groups were treated with solvent ethyl alcohol only, and the treatment groups were treated with 25, 50, 100 nM of 17β-estradiol for 12 h. Each data point represents the mean ± SEM of 6 repeats for each treatment. Different letters mean a significant difference between groups (p ≤ 0.05), and the same letter means no significant difference between groups (p > 0.05). (TIF) [file pone.0238784.s003.tif]

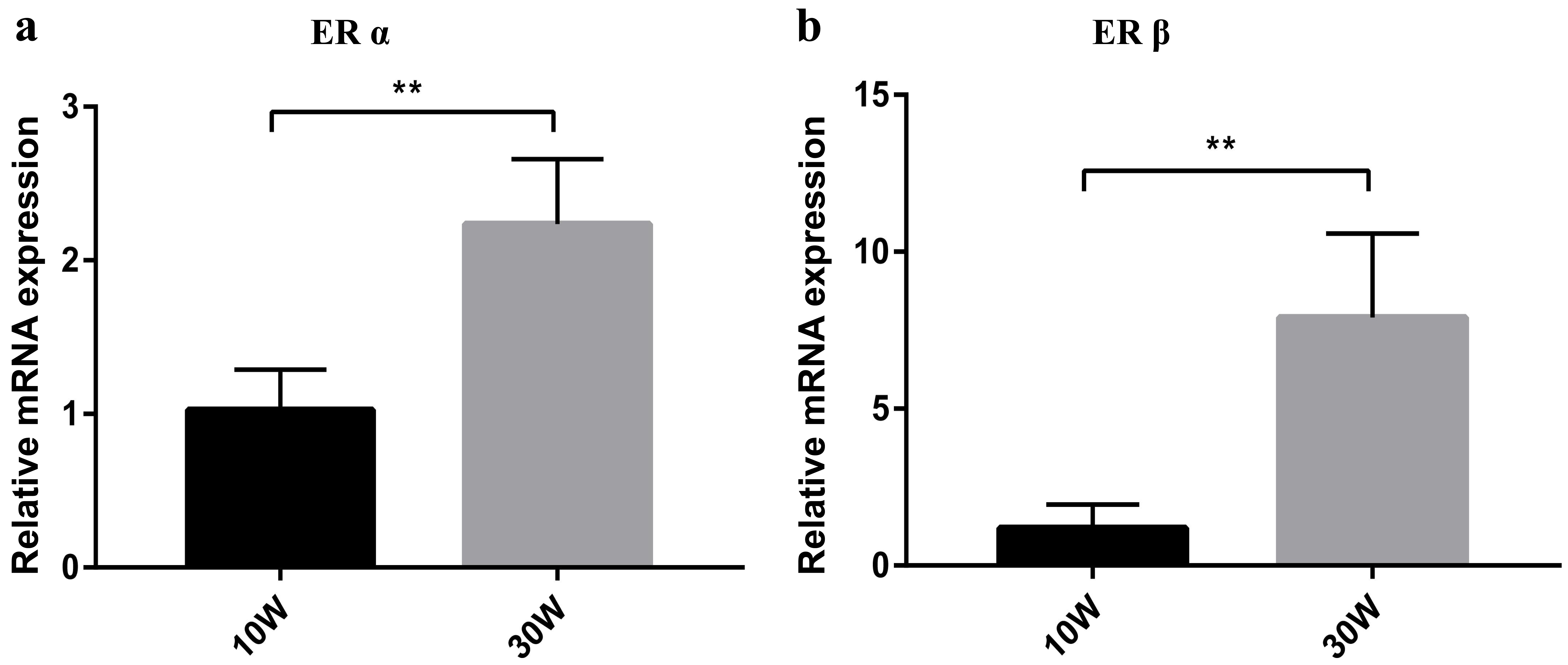

Supplement: S3 Fig — (a, b) The relative expression levels of ER α and ER β, respectively. The mRNA levels of Apelin and Apelin receptor gene was normalized to the mRNA levels of β-actin. Each data point represents the mean ± SEM of 6 chicken. Different lower-case letters mean significant difference (p ≤ 0.05), and the same lower-case letter means no significant difference (p > 0.05). (TIF) [file pone.0238784.s004.tif]

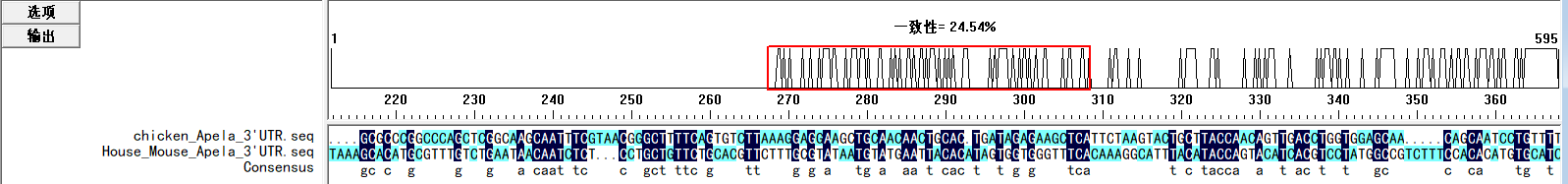

Supplement: S4 Fig — The homology analysis of the 3'UTR sequences of Apela is identical between house mouse and chicken. (TIF) [file pone.0238784.s005.tif]
